# Peripartum Anesthetic Care of a Parturient With Arnold-Chiari Malformation and Factor XI Deficiency: A Case Report

**DOI:** 10.7759/cureus.98806

**Published:** 2025-12-09

**Authors:** Devin G Monroe, Tanner Moore, Mohamad El Churafa, Taylor Varela

**Affiliations:** 1 Anesthesiology, West Virginia University School of Medicine, Morgantown, USA; 2 General Surgery, West Virginia University School of Medicine, Morgantown, USA

**Keywords:** arnold-chiari malformation, cesarean delivery, epidural, factor xi deficiency, general anesthesia, hemophilia c, neuraxial anesthesia, obstetric anesthesia, spinal anesthesia

## Abstract

Arnold-Chiari malformation (ACM) and Factor XI (FXI) deficiency are rare conditions that pose distinct anesthetic challenges in the obstetric population. While each condition has been managed individually with varying anesthetic approaches, their coexistence in a single patient significantly complicates peripartum decision-making, particularly regarding the safety of neuraxial anesthesia and the risk of hemorrhage.

We report the case of a 26-year-old G2P1011 woman at 39 weeks of gestation with ACM type I and FXI deficiency who presented for induction of labor. Multidisciplinary planning involved hematology, neurology, neurosurgery, obstetrics, and anesthesiology. Given her bleeding history, hematology recommended prophylactic fresh frozen plasma (FFP) transfusion and tranexamic acid (TXA). Due to symptomatic ACM and risk of cerebrospinal fluid leak with neuraxial anesthesia, epidural placement was deferred. Analgesia was managed with remifentanil PCA. As labor progressed, the patient elected for cesarean delivery despite increased bleeding risk. General anesthesia was chosen, and the patient received perioperative FFP and TXA. Intraoperative blood loss was estimated at 2600 milliliters, requiring transfusion of 1 unit of packed red blood cells while maintaining stable hemodynamics. Postoperatively, the patient remained hemodynamically stable with no neurologic complications and was discharged home on postoperative day five.

While neuraxial anesthesia can be considered in isolated ACM or FXI deficiency with appropriate precautions, the coexistence of both disorders amplifies the risks of neurologic deterioration and spinal hematoma. General anesthesia provided a controlled and safe alternative in this setting. This case underscores the importance of individualized anesthetic planning, multidisciplinary coordination, and careful hemostatic optimization.

The simultaneous presence of ACM and FXI deficiency in an obstetric patient presents unique anesthetic and obstetric challenges. General anesthesia with proactive hemostatic management may be the safest approach when symptomatic ACM coexists with a significant bleeding risk. This case report describes the successful multidisciplinary peripartum management of a patient with both symptomatic ACM and FXI deficiency, highlighting the anesthetic dilemma and the rationale for ultimately selecting general anesthesia for cesarean delivery.

## Introduction

Arnold-Chiari malformation (ACM) and Factor XI (FXI) deficiency, also known as hemophilia C, are two distinct medical conditions that highlight the complexities of the neurologic and hematologic systems, respectively. Each of these disease processes presents its own individual and unique challenges when providing obstetric anesthesia. 

Chiari malformations are defined as morphologic malformations that primarily involve the downward displacement of the cerebellar tonsils through the foramen magnum. There are four defined types of ACM (types I-IV) based on the degree of herniation, with type I being the most common. Those with type I ACM may be asymptomatic, and many are not diagnosed until adulthood. The most common symptom of type I ACM is headache, which is caused by increased intracranial pressure and can be precipitated by actions such as coughing, sneezing, or straining. This could be seen while straining during the second stage of labor. Other neurological symptoms of ACM include ataxia, numbness or weakness of extremities, difficulty swallowing, and nystagmus [1,2]. 

Hemophilia C, also known as FXI deficiency, is a rare genetic bleeding disorder characterized by a deficiency or dysfunction of clotting FXI, which plays a role in the intrinsic pathway of the coagulation cascade. Unlike Hemophilia A and B, Hemophilia C typically presents with mild or no spontaneous bleeding, but it can lead to excessive bleeding following surgery or trauma. This condition poses particular risks during pregnancy and delivery, especially due to the potential for postpartum hemorrhage [3-5]. Additionally, patients with Hemophilia C are at increased risk for complications with neuraxial anesthesia (such as epidurals or spinal blocks), due to the potential for spinal or epidural hematoma formation, which can result in neurological damage [3,4]. Therefore, careful pre-anesthetic assessment and multidisciplinary management are essential in pregnant individuals with Hemophilia C [3-5]. This case report describes the successful multidisciplinary peripartum management of a patient with both symptomatic ACM and FXI deficiency, highlighting the anesthetic dilemma and the rationale for ultimately selecting general anesthesia for cesarean delivery.

## Case presentation

A 26-year-old female G2P1011, 39 weeks 0 days, with a height of 165 cm, weight of 93 kg, BMI 34.2 kg/m^2^, presented to labor and delivery for scheduled induction of labor. Her past medical history is significant for Arnold-Chiari malformation type I, FXI deficiency, asthma, anxiety, and depression. Pregnancy was complicated by gestational diabetes mellitus, controlled with diet and lifestyle modifications. The patient’s surgical history includes a laparoscopic cholecystectomy, tonsillectomy, and wisdom teeth extraction. Prior to her cholecystectomy, she was given fresh frozen plasma (FFP) and did not experience any bleeding complications. She was not treated with FFP for her wisdom teeth extraction and did have excessive bleeding. This led us to believe that our patient is at an increased risk of bleeding during invasive procedures when not pretreated appropriately.

Labor was induced with oxytocin infusion, and rupture of membranes occurred on hospital admission day two. The obstetric team requested a consultation from the hematology team regarding the patient’s FXI deficiency. Labs were obtained at the time of admission, resulting in FXI activity of 62%, slightly below the normal range of 65%-150%. Other perioperative hematologic lab values are summarized in Table 1.

**Table 1:** Perioperative hematologic parameters in a patient with Arnold-Chiari malformation and Factor XI deficiency Postoperative values indicate significant anemia and coagulopathy, with prolonged prothrombin time (PT) and International Normalized Ratio (INR) reflective of dilutional/consumptive effects following intraoperative hemorrhage and transfusion. Platelet counts remained within normal limits, while activated partial thromboplastin time (aPTT) shortening is attributed to fresh frozen plasma (FFP) and antifibrinolytic therapy.

Parameter	Pre-operative	Post-operative
Hemoglobin (g/dL)	13.4	7.9
Hematocrit (%)	39.4	28.1
Platelet count (×10³/µL)	251	215
Prothrombin time (sec)	12.6	50.8
Activated partial thromboplastin time (sec)	31.4	16.6
International Normalized Ratio	1.09	4.35

The hematology recommended a transfusion of 2 units of FFP at the onset of labor. Additionally, it was recommended that the patient should receive 2 units of FFP prior to vaginal delivery, and in the case of cesarean delivery, the patient should receive 4 units of FFP. In addition to FFP, the hematology team also recommended that the patient receive 1000 mg of intravenous tranexamic acid (TXA) every 12 hours at the onset of labor. Neurology and neurosurgery teams were also consulted regarding the patient’s ACM, and no specific recommendations were made. The anesthesia team was consulted to perform a labor epidural for this patient.

**Figure 1:**
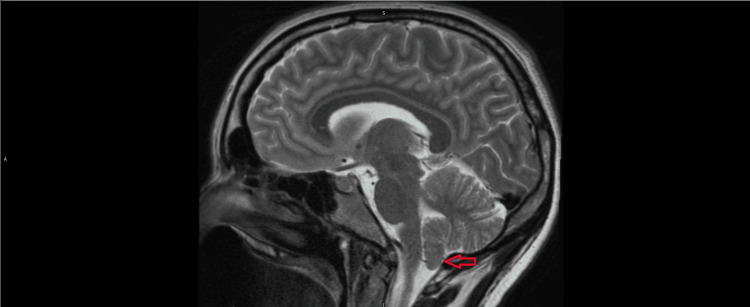
Sagittal T1-weighted MRI of the brain in a patient with Chiari Malformation Type 1 Sagittal MRI demonstrating inferior displacement of the cerebellar tonsils below the level of the foramen magnum (indicated by red arrow), consistent with Chiari Malformation Type 1. The posterior fossa structures are crowded, but there is no evidence of Syringomyelia or hydrocephalus.

The patient’s most recent MRI was reviewed, which demonstrated 4 mm of inferior descent of cerebellar tonsils below the level of the foramen magnum seen in Figure 1. Previous MRI imaging reports describe as much as 9 mm of inferior descent. After gathering a thorough history from the patient, she admitted to symptoms of increased intracranial pressure in the form of head pain when coughing or laughing. It was determined after thorough discussion with the patient that she may be at increased risk for severe neurological deficits in the event of CSF loss from intrathecal puncture during a neuraxial procedure. The patient was provided with a remifentanil patient-controlled analgesia pump set to a 2 mcg patient-controlled trigger every two minutes for pain with labor contractions. As labor progressed, the patient’s pain worsened. Due to severe pain, the patient requested to forgo vaginal delivery and pursue cesarean section delivery, even with her increased bleeding risk.

On the day of surgery, the anesthesia team prepared for a Cesarean section. General anesthesia was selected as the anesthetic of choice, given her increased risk of neuraxial anesthesia based on her history of ACM with concomitant Factor IX deficiency. Preoperatively, she received 4 units of FFP and was premedicated with diphenhydramine, acetaminophen, and hydrocortisone due to a prior reaction per recommendations of the hematology team. She was typed and crossed for an additional 4 units of packed red blood cells. General anesthesia was induced with 200 mg propofol, 140 mg succinylcholine. Once the baby was delivered, 2 mg midazolam, 100 mcg fentanyl, and 50 mg rocuronium were given along with 1000 mg TXA, and oxytocin infusion started at 334 milliunits/min with a 2-unit bolus. Anesthesia was maintained with a combination of sevoflurane, nitrous oxide. Packed red blood cell (PRBC) infusion started shortly after delivery; 1.5 L crystalloid fluid was given, 0.5 mg hydromorphone was given for pain control, and the patient was reversed with sugamadex at 4 mg/kg at the end of the procedure. Blood loss was calculated for a total of 2680 ml at the end of the procedure. The patient remained hemodynamically stable throughout, with a brief period of hypertension. Carboprost tromethamine, methylergonovine maleate, and misoprostol were also given. She remained hemodynamically stable throughout and received 1 unit of packed red blood cells intraoperatively. The patient was returned to her labor and delivery room at post-anesthesia care unit (PACU) status and moved to step-down status after a three-hour recovery period.

Postoperatively, the patient was monitored closely for signs of overt bleeding. Complete blood counts and coagulation panels were trended every six hours, with the lowest hemoglobin reaching 7.9 g/dL on post-operative day 2. She remained hemodynamically stable, and her postoperative course was overall uncomplicated. 1 Unit of FFP and 1 G TXA were given daily while hospitalized. She was eventually discharged on hospital admission day five. At that time, her TXA was transitioned to oral for a total of 21 days of therapy per the recommendation of the hematology team.

## Discussion

The use of neuraxial anesthesia (e.g., epidural or spinal anesthesia) in pregnant women with FXI deficiency has been approached with caution due to concerns about spinal or epidural hematoma formation from impaired hemostasis, which can lead to neurological complications [1-3,6]. However, multiple retrospective studies and case series indicate that, with careful patient selection and multidisciplinary management (including hematology consultation, assessment of bleeding history, and peri-procedural hemostatic optimization), neuraxial techniques can be performed safely. The risk of bleeding does not correlate strictly with FXI levels, but a personal history of bleeding is the strongest predictor of perioperative hemorrhage [3,4,6]. Most published case series report no cases of neuraxial hematoma, even among women with partial FXI deficiency undergoing labor epidural or spinal anesthesia, provided that FXI levels are above 40 IU/dL and appropriate prophylaxis (e.g., fresh frozen plasma or antifibrinolytics) is administered as indicated [3,4]. Though our patient's values were above the threshold and prophylaxis was given, the patient’s bleeding history maintained her at a high risk of epidural hematoma.

Similarly, pregnant patients with ACM require careful consideration for use of neuraxial anesthesia, as concerns exist regarding the risk of increased intracranial pressure or herniation following dural puncture [4-5]. While some studies and case reports support the safe use of neuraxial techniques in ACM patients, particularly those without severe symptoms or syringomyelia. In patients with ACM, the primary concern with neuraxial anesthesia is the potential for neurologic deterioration due to altered cerebrospinal fluid dynamics and the theoretical risk of brainstem herniation, particularly in the presence of increased intracranial pressure [4-5]. The most common complication noted is post-dural puncture headache or temporary worsening of ACM symptoms already present. However, large case series and systematic reviews demonstrate that, in the absence of clinical or radiographic signs of raised intracranial pressure, neuraxial anesthesia (including both epidural and spinal techniques) does not increase the risk of neurologic complications [4-5].

The safety of general anesthesia for cesarean section in patients with Chiari malformation has been supported by several case series and retrospective reviews, which report no increased risk of neurologic deterioration or exacerbation of Chiari-related symptoms when general anesthesia is used in the absence of signs of increased intracranial pressure [4,5]. The American Society of Anesthesiologists and the Society for Obstetric Anesthesia and Perinatology recommend that the choice of anesthetic technique for cesarean delivery should be individualized, taking into account neurologic status, obstetric indications, and patient-specific risk factors [7]. In patients with symptomatic Chiari malformation or those at risk for increased intracranial pressure, general anesthesia may be preferred to avoid the theoretical risk of brainstem herniation associated with neuraxial techniques, although the overall risk appears low in well-selected patients [3,4,6].

The presence of FXI deficiency introduces additional considerations, as this rare coagulopathy increases the risk of perioperative bleeding [3,4,7]. While neuraxial anesthesia is often preferred for cesarean delivery due to lower maternal morbidity, it may be contraindicated in patients with significant bleeding risk unless factor levels are corrected and a hematology consultation is obtained. General anesthesia is considered a safe alternative in this context, provided that appropriate perioperative hemostatic management is implemented, such as administration of fresh frozen plasma or recombinant FXI as indicated by hematologic assessment [3,4,7]. Multidisciplinary planning involving anesthesiology, hematology, and obstetrics is essential to optimize outcomes and minimize complications in patients with both Chiari malformation and FXI deficiency undergoing cesarean section.

## Conclusions

While both Arnold-Chiari malformation (ACM) and Factor XI (FXI) deficiency have demonstrated some degree of safety with neuraxial anesthesia when considered individually, the coexistence of these two conditions significantly increases the potential risk to the patient. Our literature review revealed no reported cases of neuraxial anesthesia being performed in patients with symptomatic manifestations of both disorders concurrently, highlighting a critical gap in evidence and reinforcing the need for caution. In contrast, a planned general anesthetic in a controlled setting with a stable patient offers a more predictable and safer alternative. As with other complex clinical scenarios, a thorough risk-benefit analysis should be conducted by a multidisciplinary team in close collaboration with the patient. Notably, proper patient selection is a recurrent theme in the literature and remains a key determinant in ensuring safe anesthetic care. This case suggests that in parturients with ACM and a bleeding disorder, a planned, multidisciplinary approach utilizing general anesthesia may represent the safest strategy to mitigate potentially catastrophic complications.
